# Collectanea

**Published:** 1831-02

**Authors:** 


### COLLECTANEA.

**Floriferis ut apes in saltibus omnia libant,

Omnia nos, itidem, depascimur aurea dicta.**

### PATHOLOGY.

*Pellagra at Milan.* The hospital contained a considerable number of patients suffering from pellagra, that well-known endemic of Upper Italy, which is generally supposed (and in Dr. Lucca's opinion also,) to arise from bad provisions, especially polenta, and the bad bread of the country people. Dr. Lucca thought, moreover, that this condition of the system essentially depended on a gastro-enteritis, caused by these bad provisions; a theory which is, perhaps, too much in the taste of the modern French school. I should rather suppose, (if a mere cursory observer may be allowed to give his opinion,) that uncleanliness and wretchedness produce a diseased mixture and movement, and even chronic inflammation in the lymphatic glands and vessels, in short, a modification of scrofula, showing itself in a peculiar disease of the skin, which at last becomes fatal by undermining the general powers of life.—*Analecten zur Naturwissenschaft und Heilkunde. Gesammelt auf einer Reise durch Italien, im Jahre* 1828. Von Dr. Carl Gustav Carus. Dresden, 1829.

---

*Collection of Fæces in the Intestines, producing Symptoms of internal Strangulation. Croton Oil applied with success upon a blistered Surface.* A man was attacked, after a voyage, with symptoms of internal strangulation; he vomited incessantly; bowels constipated; belly swollen, and very tender upon pressure; fecal matter had been thrown from the stomach. The patient had had a reducible hernia for a long time, but he affirmed that nothing particular had occurred as to his rupture: he wore his truss as usual. Warm baths, repeated bleeding, &c. were tried, without any benefit. A consultation was held to determine if this was a case of strangulated hernia, and whether an operation should be performed? The majority of consultants advised that nothing should be done!

The state of the patient still remained the same: continued fecal vomiting, and his strength declining. Up to the fifteenth day, no change had occurred. A celebrated surgeon was now called in; and he concluded, from the unequivocal symptoms of strangulation, that an operation was demanded, and he advised its performance, although there were no external signs of strangulated hernia, either in the inguinal canal, in the abdomen, or near the ring. M. Sanson, therefore, retired to prepare for the operation. He was about to commence it, when he was induced to hesitate, as he could detect neither tumor, external pain, nor tension, above the ring, or in the inguinal canal, or deep-seated in the neighbouring parts. That part of the abdomen, indeed, which corresponded to the hernia was the only part of it which was not painful and tense. Upon very carefully examining the whole surface of the abdomen, he felt, upon the left side, a deep-seated and lengthened swelling, in the form of a column. He imagined it was the colon, filled with hardened fæces. He carried his finger as high up the rectum as he could: in doing this he had great difficulty, as the sphincter ani was much contracted. M. Sanson then endeavoured to introduce an elastic-gum sound, but he could pass it but a very short distance, so powerful was the constriction of the intestine. Several clysters of olive oil were thrown in with considerable force. The first had no effect; but, by continuing them, some soft, yellowish matter was brought away. They were continued uninterruptedly. Purgatives could not be administered by the mouth, as the vomiting was so incessant that the patient could not even retain upon his stomach a spoonful of water. A small blister was applied to the thigh, and, when the epidermis was removed, one drop of croton oil was placed on the abraded surface. An abundant evacuation from the bowels soon took place; the vomiting ceased; several pounds of fecal matter, soft and yellow, were voided in the course of three or four days, and all the symptoms subsided, and it was very evident that they had arisen entirely from fecal accumulation in the intestinal canal.—*Journal der Med. Prat.*

---

*Case of Poisoning with Corrosive Sublimate, in which the Administration of Charcoal afforded great relief.* By WILLIAM P. HORT, M.D. of Bladen county, North Carolina.

August 4th, 1829. John Hidleston, a stout man, of robust constitution, about forty years of age, finding himself much indisposed, and supposing that he was very bilious, determined to take a dose of tartar emetic. Under the impression that the strength of his tartar emetic had become impaired, an idea which is very prevalent in the country, he resolved that the dose should be unusually large, and dissolved nearly a tea-spoonful of tartar emetic, as he supposed, in a cup of warm water: of this solution he drank one half, and waited for the emetic operation. In about fifteen minutes he was alarmed by a burning sensation increasing rapidly in intensity, and extending gradually from the mouth to the stomach. On examination, he ascertained that he had taken corrosive sublimate instead of tartar emetic, and immediately sent for me; but I was unfortunately absent at the time, and did not see him until the next morning. On hearing that I was from home, he applied for advice to one of his neighbours, who administered a large dose of Epsom salt about three hours after he had swallowed the solution of corrosive sublimate. When I saw him on the 5th of August, early in the morning, his situation was extremely distressing; he complained of intense burning pain throughont the whole course of the alimentary canal; and the contracted state of the muscles of his face, and general appearance, clearly evinced the severity of his suffering; his skin was cold and clammy; his pulse small, hard, and increased in frequency; he complained of urgent thirst, but had not dared to drink any thing to allay it. The dose of salt taken the day before had operated promptly and violently, producing tormina, tenesmus, and bloody and fœtid stools. His physical energies were completely prostrated, and his mind was very irritable. He was bled copiously, probably to the extent of three pints, and I directed him to make free use of the white of eggs, beat up with sugar. On the 6th, in the morning, he was worse: his bowels were in constant action; the discharges were still bloody, and very offensive; his skin was cold and clammy; the pulse very feeble and frequent He said the pain in the stomach and bowels was almost past endurance, and observed that the prescription of the white of eggs and sugar had produced distressing nausea, and no mitigation whatever of his sufferings.

Convinced that the violent inflammation of the bowels was verging fast towards gangrene, and that the state of extreme debility, to which the constant purging and pain had reduced him, prohibited any further depletion, I ordered a tea-spoonful of finely pulverized charcoal to be administered in gruel every hour, and left him, scarcely entertaining a hope of seeing him alive on the morrow. The next day, August 7th, I called on my unfortunate patient about ten, A.M. and to my great surprise, on approaching his bed, was greeted with a smile; his first expression was, that the charcoal had saved his life. He assured me that shortly after taking the first dose, he had experienced very sensible relief, and that he perceived a regular alleviation of pain after every succeeding dose. He was evidently much better, and unexpectedly rescued from the grave. I directed him to continue the use of the charcoal, gradually increasing the intervals between the doses for several days: his convalescence was steady, though slow; the purging continued until the tenth day; and several months elapsed before the tone of his stomach and bowels was restored.

A considerable portion of the solution of the corrosive sublimate was undoubtedly carried off by the operation of the salt, or the man must inevitably have perished, as the antidote for the poison was not administered until twenty hours had elapsed; in which time so large a quantity of corrosive sublimate, as he swallowed, would have produced an extent and degree of inflammatory action, which subsequent treatment could neither have subdued nor palliated. When he commenced the use of the charcoal, he was evidently sinking rapidly, and the case appeared quite hopeless. I have since questioned him closely on the subject, and he affirms most positively that the charcoal saved his life, and that he could not be mistaken in the effects of its operation.—*American Journal of Med. Sciences.*

PRACTICAL MEDICINE.

*Belladonna in Hooping Cough.* Much has been said, both for and against the use of this medicine. We believe that most practitioners feel the want of some remedy which is capable of relieving the violent paroxysms of coughing, which often continue for a long period after the first and inflammatory stage of the disease has passed away. Dr. MIGUEL, of Neuerhaus, places much confidence in belladonna in hooping-cough. In the course of several epidemics, which he has observed during the last fifteen years, he has constantly, as he assures us, removed the cough in eight days by its assistance. He has administered it, however, with certain precautions. Before having recourse to it, plethora or gastric derangement must be combated. M. Miguel gives the belladonna from the commencement of the disease, if neither of these contra-indications exist. His experience has taught him that it should be given in progressive doses, until signs of narcotism (agitation and redness of face,) begin to be manifested: then, without discontinuing the medicine, he diminishes the doses of it, so that each dose may produce a slight agitation for about an hour.

According to this physician, no remedy so quickly loses its efficacy by keeping as belladonna. When the root has been gathered a year, two thirds of a grain may be given three times a day, to children of two years of age, without any remarkable effect. The fresh root, on the contrary, acts very sensibly in the dose of one eighth of a grain. Thus M. Miguel has known this dose of the fresh root (in May) produce agitation and redness of face, while, when it had been properly kept until the December following, it produced no effect in the dose of half a grain in a child of the same age.—(*Archiv. fur Mediz. Erfahrung*, 4 cah. p. 578.)

---

*Endermic Method of applying Acetate of Morphia in Delirium Tremens.*

CASE I. A patient named Harrigan was admitted into the Pennsylvania Hospital in December last, with delirium tremens: he had been ill several days, and had not slept since the commencement of the attack. It being necessary to administer opiates, the acetate of morphia was directed; but the extreme gastric irritability of the patient rendered it impossible for him to retain it a moment; and all the other remedies which were prescribed failed to allay the nausea. The patient was now sinking, when a blister was applied to the epigastrium, and six grains of the acetate of morphia sprinkled upon the surface, about five o'clock, P.M.; the patient was rather relieved and slept about an hour. The next day four grains were applied to the blister, the patient continuing quiet, but with very little sleep: and on the third day another application of five grains was made, which produced the desired effect; the patient obtaining a quiet sleep of several hours, followed by complete recovery. I am indebted to Dr. HAMERSLY for this case, which is peculiarly interesting, from the fact that the patient could scarcely have escaped death without resorting to endermic medication.

Case II. Moses Decker, aged twenty-seven. Very intemperate. Admitted May 8th, 1830, but was not attacked with delirium tremens until the 10th. He had been purged, &c.: had slept, but not longer than three or four hours at one time.

10th. Pulse 104, tongue white and furred at the centre, rather red at the edges. Dr. Jackson saw him in the morning, and directed his head to be shaved, and bladders filled with ice to be constantly kept upon it, with warm applications to his feet. He was also cupped freely on the head, and a blister six inches square applied to the epigastrium; it was dressed at half past nine p.m. with a powder composed of sulph. quinine gr. iv. sulph. morphia gr. ij. M.

He was at this time very delirious, crying out that he should die; imagining that every one had designs upon his life. At twelve o'clock, being still much excited, another powder was applied.

11th. Slept three hours and a half last night; is more rational, but still timorous; pulse ninety, rather feeble; tongue has cleaned; bowels open. Another powder applied at seven o'clock, but omitting the quinine; slept an hour or two in the morning. Awoke at half past twelve, when his blister was again dressed with a powder composed as the last, of two grains of morphia. Fell asleep a few minutes after the dressing, and slept more than six hours. Awoke at seven p.m. entirely rational; attempted to eat some indigestible food which he had obtained, but his stomach was still too weak to receive it. Another powder of the morphia was applied; slept within an hour afterwards, and rested well during the night. The next day his somnolency continued, sleeping at least eighteen hours in the twenty-four, but perfectly rational when awake. No medicine was taken internally during the disease, with the exception of an effervescing draught to allay the accidental nausea, and one or two glasses of porter. The patient now continued entirely convalescent, and was very soon discharged well: the whole quantity of the sulphate of morphia necessary for the cure was twelve grains.

Without any pretensions to an infallible remedy for a disease so extremely uncertain and liable to complication as delirium tremens, which the observation of a large number of patients in an institution, where a greater number of cases are undoubtedly treated than in any other, has convinced us, will often baffle the efforts of the most skilful practitioners; it will not, I trust, appear presumptuous to recommend this method as of immense utility in a great number of cases, as an adjuvant, and, in not a few, as the mode of treatment principally to be relied upon for success. In several other instances the method of treating the disease was pursued; occasionally without the addition of internal remedies, but more frequently as an adjuvant, and the results have been very satisfactory when compared with the usual methods.—(Dr. Gerhard, *North American Med. and Surg. Journal.*)

*Endermic Application of Cathartics and Diuretics.* One of the most interesting, if not the most useful facts furnished by endermic medication, is the ready and powerful action of some of the cathartics. The external use of narcotics has long been familiarly known in certain diseases, and the action of tonics may be readily supposed, independent of the tissue to which they are applied; but catharsis seems so intimately connected with a direct stimulation of the intestinal membrane by our remedies, that most practitioners would scarcely credit the facility with which it follows an endermic application. Although many of the purgatives operate readily in this manner, yet it must by no means be imagined that all substances of the class are equally manageable; the great variety of their chemical composition renders a selection very necessary to a successful result. As in the other classes of remedial agents, those from the vegetable kingdom are alone included in our list; while solubility in the animal fluids, concentration without very acrid properties, and the other requisites before explained, must always be sought for in the articles to be employed. A necessity for the present mode of exhibiting cathartics rarely exists; but still in the case of maniacs, perhaps in children, although I cannot speak from any experience of endermic medication in their diseases, and other cases which every practitioner may occasionally meet with, it may prove useful.

*Aloes.* This medicine is the purgative best adapted for endermic medication; it does not irritate powerfully, is very soluble, and acts in nearly the same dose as when taken internally. The dose is ten grains, repeated, if necessary, in a few hours.

Case I. William Young, aged forty. A blistered surface upon the epigastrium was dressed with ten grains of powdered aloes, which was repeated in the evening.

October 10th. Bowels were costive before the application of yesterday; but were last night opened six or seven times with griping and watery stools. The first evacuation occurred about an hour after the application of the aloes, and was preceded by a sensation of heat apparently arising from the part to which it was applied. Ten grains sprinkled upon the blister in the morning, produced five or six discharges during the day.

11th. Bowels still freely open; purgative discontinued.

Case II. John Lynch, aged thirty-nine. October 9th. Ten grains of aloes were sprinkled upon a blister at the epigastrium; bowels costive.

10th. Well purged this morning, with griping: two applications were made during the day: he was purged five or six times.. He experiences no heat in the abdomen, and complains of no pain from the dressings.

Case III. Owen Scolly, aged thirty. The bowels of this patient being very costive, a blister on the epigastrium was dressed with the following cerate: Pulv. aloes socot. gr. xxiv.; cerat. simp. ℥i.

M.; one third of this quantity to be applied three times a day. By this the patient was freely purged.

Case IV. George Comly, aged forty-two. He was afflicted with obstinate headach, attended with constipation; it had been for some time found necessary to purge him with twelve grains of aloes every other day; the same quantity was now sprinkled upon a blistered surface which had been kept open on the back of the neck. Catharsis, as free as was usually produced by the same medicine internally administered, followed this application.

*Gamboge.* This article, owing to its solubility, purges freely when applied in the endermic method; it cannot. however, be used so frequently as aloes, on account of its more acrid properties.

Case V. Christopher Murdock, aged fifty-four. A blister had been applied to the back of the neck, to remove a violent headach; bowels constipated; the surface of the blister was dressed with a cerate composed of pulv. gamboge gr. xviij.; cerat. simp. ℥ss. M.; to be used in three dressings. Bowels were, in consequence, opened very frequently, with severe griping.

Gamboge and aloes, in the proportion of two or three grains of each, operated well as a laxative, when applied to the skin.

*Rhubarb.* I fairly tried this article in one instance upon the epigastrium, in the quantity of eighty grains during the day; it was continued for two days without success. It was not irritating, and was probably not efficient, merely from its want of energy.

*Jalap.* In two instances this medicine was used without any satisfactory result; it produced considerable irritation, and, being insoluble in aqueous menstrua, could not be absorbed.

*Elaterium.* The preparation of Clutterbuck, in doses of a grain, sprinkled upon the epigastrium, at intervals of one or two hours, produced merely a local action.

*Croton Oil.* I had expected a powerful operation from this medicine; but neither on blisters, nor on ulcers, even when it was increased to ten minims, was any catharsis observed.

*Compound Extract of Colocynth.* This is readily applied when softened; and in doses of a scruple repeated, it proved cathartic, but in a feeble degree.

Diuretics. The difficulties which sometimes attend the exhibition of diurectics, and the uncertainty of their operation, are subjects of general regret among practitioners. It is frequently found that dropsical effusions are attended by various grades of inflammation of the primæ viæ, and as no stimulant so active as most of this class can be tolerated in such cases, another surface for their application must be sought for: in some instances the iatraleptic method has been successful; at least it is still frequently resorted to in the Parisian hospitals, but it is at best very feeble, and far inferior to the more efficient mode of addressing the remedy to the denuded cutis. All the vegetable diurectics may be administered as endermic remedies, and in many cases the mercurials may be given in the usual mode, as an adjuvant to their action. The abdomen is the most proper place for this application, especially as it is often selected as the situation upon which blisters act with the most happy effect. The general rules for the administration of diuretics are similar to those already laid down in reference to the other remedies; the success of this class of remedial agents employed in the endermic method, in a few instances, has only induced me to regret that I was unable to test their powers by a more extended course of observation.

*Scilla Maritima.* The squill I have used separately and in combination with digitalis.

Case VI. Mary Burk, aged sixty. This patient was a long time in the infirmary, affected with ascites. A blister was applied on the 15th of October, to remove a violent rheumatic pain at the inside of the thigh; upon its removal, four grains of powdered squill were sprinkled upon the denuded surface three times a day; an abundant secretion of urine, causing the entire absorption of the fluid effused, continued until the suspension of the medicine,

Case VII. Ann Smith, aged thirty-six years, admitted October 24th, after a confinement of some weeks in prison, where she had been treated for hydrothorax. She was then labouring under general dropsy, with a frequent and rather feeble pulse, bowels regular, but urine scanty: a gill of gin, much diluted, was directed as a stimulant, to be taken during the night.

25th. Rather better, pulse fuller, diuresis more abundant during the night; a blister was directed to the abdomen, to be dressed in the evening, with pulv. scill. gr. vj.; pulv. fol. digital. gr. j. M.; to be repeated the next morning.

26th. Urine has become very copious, blister has been twice dressed with the powder; anasarca diminishing.

27th. Powder applied twice a day; urine continues abundant; the water is now entirely evacuated, and the prescription consequently discontinued.—(Dr. Gerhard, *ibid.*)

---

### SURGERY.

### *On the dangerous and fatal Effects arising from the Admission of Air into the Veins, during the Performance of Operations in the Neck.*

In the last volume of the Med.-Chir. Trans. (vol. xvi. p. 1,) Mr. Barlow directs the attention of surgeons to this important, and hitherto somewhat neglected, subject. His experience convinces him that, whatever may be the characters of tumors, when attached to important structures of the neck, whether in their incipient or advanced stage, that their excision is much more dangerous than those which are more distant from the heart. In attempting to dissect down to the extensive basis of a tumor seated on the side of the neck, Mr. B. states, that "a sudden and unexpected hissing gurgling noise rushed obviously from a large divided empty vein,* and the patient expired instantly, without either sigh, groan, or struggle; and every effort used to restore animation was fruitless. This circumstance occurred thirty years ago. Scarcely an ounce of blood was lost during the operation, and the death of the patient was attributed to a state of debility and syncope.

Mr. Barlow remained of this opinion until he met with an analogous case published by DUPUYTREN. In this instance an operation was performed for the removal of a tumor from the posterior and lateral part of the neck. No arteries were cut that required the ligature, and consequently there was very little bleeding; neither were there any muscles or large nerves divided. Just, however, as M. Dupuytren was proceeding to separate the last shreds of attachment, and turn the tumor out, he was surprised to hear a somewhat prolonged hissing noise, similar to that produced by the re-entrance of air into a vessel from which it had been exhausted. Almost instantly the patient cried out that she was dying, and in a moment dropped dead on the floor. The body was carefully dissected, but no morbid structure was found. An examination of the heart disclosed the cause of the melancholy catastrophe.†

"The right auricle was distended like a bladder with air, which rushed out when cut open, without any admixture of blood. Fluid blood was found in the other cavities of the heart, as also in the different vessels. Great quantities of air were found in all the vessels. There was no other unnatural appearance in any part of the body."

"On this important case, the editor of the Medical and Chirurgical Review observes, 'We have not the smallest doubt, that Dupuytren was perfectly correct in his conclusion, that air had rushed in through one of the veins of the neck, and thus caused instant death. We think it a pathological fact, however, which bears on the physiology of the circulation.

"It proves that the heart acts as a sucking, as well as a forcing pump, otherwise air could never have passed from a cut vein in the neck down into the right chambers of the heart.

"It is highly probable, that, in consequence of the morbid state of the parts, the mouth of the cut vein had remained patulous, and thus readily admitted the air."

This state of the incised vein was exactly similar to that in the case above mentioned by Mr. Barlow.

* "It has been noticed by authors, that the veins about the neck are sometimes found irregularly distributed, and whether the incised vein, in this case, was the external jugular, or an anomalous variety, is not easy to say; for its caliber much exceeded that vessel in its natural state, and appeared flabby and empty. The instant the atmospheric air gained access, and filled the vacuum, the hissing noise ceased, the patient expired, and the mouth of the vessel collapsed."

† At page 122 of this number, we have related another, and very similar, case, which occurred in a Parisian hospital.—EDITOR.

Another case of a similar kind has been recorded by Professor Mott, of New York: he says,

"In an attempt which I made to remove the parotid gland in an enlarged and scirrhous state, the facial vein, where it passes over the base of the lower jaw, was opened in dissecting the integuments from the tumor, in the early stage of the operation, before a single artery was tied. At the instant this vessel was opened, the attention of all present was arrested by the gurgling noise of air passing into some small opening.

"The breathing of the patient immediately became difficult and laborious, the heart beat violently and irregularly, his features were distorted, and convulsions of the whole body soon followed to so great an extent as to make it impossible to keep him on the table. He lay upon the floor in this condition for near half an hour, as all supposed in articulo mortis.

"As the convulsions gradually left him, his mouth was permanently distorted, and complete hemiplegia was found to have ensued; an hour or more elapsed before he could articulate, and it was nearly a whole day before he recovered the use of his arm and leg. From a belief that these effects arose from the admission of air into the blood-vessels, which was not doubted by any person present, I instantly called to mind a set of experiments which I made some twenty years since upon dogs, by blowing air into the circulation, by inserting a blow-pipe into a large superficial vein upon the thigh, and was forcibly struck with the similarity of result."

Dr. Blundell* relates some experiments of the introduction of air into the blood-vessels of dogs, from which, and the above cases, it appears that the entrance of even a few drachms, though introduced at a distance from the heart, and whilst the animals were in perfect health, eventually produced considerable distress and danger. "Hence," says Mr. Barlow, "we may conclude, when extirpating morbid tumors seated about the neck, where the veins are frequently enlarged and superficial, and the parts subject to organic derangement of structure, and the patient at the same time labouring under a state of mental anxiety and constitutional debility; that, under these unfavorable circumstances, if a large vein be opened and syncope ensue, the danger becomes manifestly alarming; and to avert such incidents, where there is reason to anticipate mischief, would it not be prudent on the part of the surgeon, when commencing such operations, either to apply pressure on the visible veins which appear in the way of the knife, or secure them by one or two ligatures, either effectually or only during the time of the operation? whereby the operator would be secured, from fear and the patient protected from danger."

A case similar to those mentioned by Mr. Barlow occurred at one of the London hospitals some time ago. A man was bled in the jugular vein, and when the blood ceased to flow, and com-

* Researches, Physiological and Pathological, p. 131.

pression was suspended below the puncture, a hissing sound was heard, as if air entered the vein. The patient immediately struggled violently, and almost instantly fell dead.

In the London Medical Gazette, July 19th, 1828, a case is related, in which M. Bouley, a very able veterinary surgeon of Paris, bled a horse, labouring under pneumonia, with the phleme, in the usual way. Compression upon the vein below the puncture was suspended for a moment, and at the same instant a remarkable noise was heard. The animal was soon affected with general trembling, and laborious breathing; he uttered some deep groans, and fell "as if stricken by lightning." M. Bouley believed that the noise he had heard, arose from the rushing of air into the vein, and he instantly determined to draw more blood from the animal. As the blood flowed, the horse "appeared to assume new life," and soon recovered.

Professor Dupuy, of Alfort, witnessed a similar accident, in which a second bleeding was also immediately effected. The case terminated favorably.

These facts shew the necessity of always very carefully closing the wound made in bleeding from the jugular vein, before the pressure below it is removed. M. Dieffenbach (London Med. and Phys. Journal, February 1830, p. 183,) made some experiments upon this point. He propelled into the jugular vein of a large cock, two years old, as much air as he was able at one continued expiration: the animal made a loud noise, and fell dead. The pulsation of the heart ceased immediately after the introduction of the air; the pupils were much dilated. When small portions of the comb were cut off, a quantity of frothy blood issued from the little wounds. Bubbles of air adhered to the comb. Air was also detected between the laminæ of the mesentery, which were raised up like globular vesicles.

M. Magendie published (Journal de Physiologie, Avril 1821,*) a memoir upon the accidental entrance of air into the veins, and upon the sudden death which follows. He states that, when a certain quantity of air is quickly introduced into a vein, it causes immediate death, because it accumulates and is rarefied in the cavities of the heart: their contraction is thus prevented, and the circulation of the blood ceases. His opinion is founded principally upon the following fact. A man had a large tumor above the right clavicle. During the operation for its removal, he suddenly cried out, "My blood stops in my body; I am dying;" and at the same moment he became perfectly stiff and insensible, and was covered with cold sweat. A remarkable and loud noise had been heard, as if something was passing into the interior of the chest. The patient died in forty-five minutes after the commencement of the operation. The body was examined the next morning. In the

* The substance of this paper is given in the Dict. des Sc. Med. tom. 57, p. 137.—Editor.

chest nothing peculiar was found; but there was an opening in the jugular vein, just where it enters the subclavian. Bubbles of air were observed in the vessels of the brain.

To relieve the dangerous symptoms which arise from the introduction of air into the veins of animals, Magendie has adopted the following plan: When air has entered the jugular vein, and the hissing noise is heard, he passes a silver tube into the vein towards the heart, and even into the right auricle. A syringe is then adapted to the extremity of the tube, and the air and blood contained in the auricle are drawn out. The syringe is then withdrawn and emptied, and the same proceeding is repeated. By this means, he says that the noise, which arises from the agitation of the air in the heart, ceases, and the circulation again goes on. He suggests the same expedient in the human subject; but it would appear from the above facts, that there is rarely time for its application. To prevent similar accidents in the practice of surgery, he recommends surgeons carefully to avoid wounding the veins of the neck in operations, and if unfortunately they are wounded, the lower end of the vessel should be tied.

We may just mention one or two facts connected with this subject, for which we are indebted to our very zealous and intelligent friend, Mr. FIELD, jun. the veterinary surgeon. In bleeding horses in the neck, the rushing noise above described is frequently heard, and the animal suffers afterwards great distress in breathing; but he has never known death to follow from the admission of air in this manner. The necessity of continuing the pressure below the opening in the jugular vein, until it is carefully closed, in order to prevent the entrance of air, has long been known to veterinary surgeons. Mr. Field has occasionally destroyed horses by blowing air into the veins; but he states, that a very considerable quantity must be thrown in to kill the animal. We recently saw an ass destroyed in this manner by Mr. Field: the jugular vein was opened in the usual way with a phleme, and compression below the wound was removed, in order to ascertain whether the rushing sound from the admission of air would be heard. No such sound, however, was detected, nor was there any reason, from the appearance of the animal, to believe that air entered the vein. Mr. Field then introduced a tube into the vein, and blew air in forcibly. In two seconds the animal dropped: the breathing was very laborious, and rather slower than natural. After a few weak struggles, during which the limbs were slowly extended, the animal died, about two minutes after the introduction of air. The chest was examined: from all the superficial veins of the neck, bubbles of air and frothy blood escaped, when they were divided. From the appearance and feel of the heart, it was evident that it was much distended with air. The right ventricle was opened, and the heart immediately collapsed; a considerable quantity of air, mixed with blood, rushed out at the same moment. Several of the above facts very strongly confirm the ingenious physiological deductions of Dr. Barry.*

Butchers always take blood from the jugular vein of calves, a day or two before they are slaughtered, for the purpose of giving the flesh a white and delicate appearance. They first apply a ligature round the neck "to plump the vein;" an incision is then made with a phleme, and blood is drawn till the animal faints. The ligature is removed, and no attention whatever is paid to the wound. We are informed by a wholesale butcher, who has slaughtered some thousand calves, that the animals always recover from this careless bleeding in the jugular vein, and that secondary bleeding never arises. He had never heard of danger from the admission of air into the vein. It is probable that the thickness of their hide, and its slight disposition to retract, prevent the admission of air into the wound, and protect these animals from the accidents and severe symptoms which not unfrequently arise from opening the veins of other animals that have a more delicate and retractile skin.—Editor.

---

*Amputation of the Leg at the Knee-joint.* M. Velpeau lately read a paper upon this operation, at the Académie des Sciences. He does not consider it so dangerous as it is generally deemed, and he even thinks it ought to be preferred to the common mode of amputating, where the joint is healthy. He has twice performed it with success. In the first case, the patient, a young man, had necrosis of the tibia, a part of which M. V. intended to remove; but, after having made a transverse incision, the bone was found to be diseased throughout. It was necessary, therefore, either to amputate the thigh, or to have recourse to disarticulation of the leg. The latter was preferred. Nothing remarkable occurred during the operation. Complete union of the wound quickly followed, and the patient was quite well in about two months.

In the second case, a man had fractured his left leg. He was taken to the Hôpital St. Antoine, twenty-four hours after the accident: considerable bleeding had occurred, and nearly the whole of the limb was in a state of ecchymosis. The progress of the case was very unfavorable, and it was at length evident that the limb must be removed, without further delay. Amputation below the knee was not practicable in this instance, and M. V. determined to perform the disarticulation of the leg at the knee-joint. This operation was performed on the 4th of June; and on the sixtieth day the patient was cured.

In another case, which M. V. had seen, the same operation had been performed, with success, upon a child, ætat twelve. Besides these cases, others have been recorded by surgical writers; and M.

* Experimental Researches on the Influence of Atmospheric Pressure upon the Blood in the Veins, &c. By David Barry, m.d.

Velpeau concludes, that disarticulation of the leg ought not to be entirely banished from the list of surgical operations, although most modern authorities are opposed to it.

Baron Larrey has made a report to the Institute Royal, upon the practice recommended by M. Velpeau, of removing the leg at the knee-joint, when the condyles of the femur are not diseased. The Baron is not inclined to extend the operation to acute affections; but he believes it may be adopted with success in cases of necrosis and gangrene, when the limits of the disease are defined, as well as in most chronic diseases of the leg, which do not implicate the upper and articular portion of the tibia.

---

*On the Cauterization of the Transparent Cornea in Diseases of the Eye, attended with Dilatation of the Pupil.* Read at the Atheneum of Medicine, by M. Serres.

Cauterization of the transparent cornea in cases of mydriasis has been too much neglected, because it has not always been successful; and we should scarcely venture now to mention this practice, if we had not witnessed its advantages, and also seen some unfortunate hospital patients who had been abandoned to their misfortune, when they probably might have been restored to sight.

Case I. Louis Perrin, æt. twenty-five, an artillery soldier, received a blow on the eyeball with a piece of wood. He was knocked down, and immediately deprived of the power of distinguishing objects. The eye became very painful and inflamed: he continued his route, however, and in ten days arrived at Montpellier. The pain had now nearly subsided; but, if he endeavoured to look at any object in a strong light, the eye filled with tears, and his vision was very imperfect. The pupil was so much dilated as nearly to prevent the iris from being visible. He was bled, and a moxa was applied to the right temple, without much benefit. *The cornea was now cauterized in different points of its circumference, and the pupil almost immediately contracted.* Inflammation followed, either from the conjunctiva being touched, or from the too great strength of the caustic; but it was soon relieved by appropriate means. The sloughs produced by the cauterization were gradually detached, and the cornea recovered its transparency. *The pupil, although still contracted, became more and more accessible to the rays of light, and the patient was conscious of an improvement in his sight.* About a month from the first application of the caustic, he saw well enough to read very small print, at a foot and a half distance, and he quitted the hospital.

*Remarks.* This case, the first of the kind we had observed, was quite sufficient to attract attention. It is worthy of observation, that the pupil contracted the moment the caustic was applied. Now, if the iris is so sensible to the application of caustic to the cornea, and if it only contracts under the influence of the retina, it must be presumed, that the action of the nitrate of silver extends to the retina. Some good had indeed been done by the moxa, but the cauterization of the cornea augmented the improvement. The success of this treatment may be thus accounted for: the relation between the ciliary ganglion and the cornea offers as available an explanation as that derived from the connexion of the temporal or obitar nerves with the nervous apparatus of the globe of the eye.

Case II. M. L***, a medical student, was struck with a stone just between the eyebrow, and opposite the superciliary foramen. He was instantly deprived of the sight of both eyes, and was led home. In a few hours, the pupil of the eye which had been struck was excessively dilated. M. L. suffered much during the night, and in the morning he was bled from the arm. The following day, leeches were applied behind the mastoid processes. Emollient lotions, foot baths, with mustard clysters and laxative ptisans, were used. As these remedies only diminished the inflammation, it was determined to cauterize the cornea, with the view of exciting the sensibility of the iris and retina. The iris contracted, but vision was not restored. From a second use of the caustic, a good deal of inflammation was produced. The sight gradually amended, but the patient, being rather impatient, consulted Prof. Lallemand, who recommended electricity. The pupil very nearly recovered its ordinary dimensions, and there were no traces on the cornea of the cauterization. M. L. saw enough to read tolerably well even with the injured eye, but he occasionally saw objects double, or in a reversed position.

*Remarks.* In this case the cure was incomplete, but still the influence produced by cauterizing the cornea was evident. It may be presumed, perhaps, that electricity was here the active agent in the improvement of sight; but M. Demours, who has often tried it in cases of mydriasis, asserts that the benefit which arises from it is but momentary, and that, when he employed it, the pupil, in a few minutes, was as much dilated as before, and the patient no longer able to read. The slowness with which vision was improved in this instance, by shewing the severity of the injury, proves the power of the remedy. It is very clear that we had not here to contend with simple and temporary dilatation of the pupil. That the retina must have sustained considerable injury, is proved by the imperfect manner in which its functions are still performed. The inflammation which succeeds the application of the caustic, is not of frequent occurrence, and is usually slight, if care be taken that the cornea alone is touched, and only upon some points of its circumference. In this case, it was necessary that the whole circumference of the cornea should be cauterized, and it was therefore unlikely that the conjunctiva should not be affected by the nitrate of silver.

Case III. An old soldier, of the name of Hogde, after much fatigue and exposure to severe weather, was attacked with a sudden dizziness and loss of sight of the right eye. Vision was soon restored, but from this time the eye grew daily weaker. The slightest cause, as sneezing, for example, obscured his sight. The right eye was principally affected; and, towards dusk in the evening, he could with difficulty see his way. The pupil was considerably dilated. Such was the state of the man when he was admitted into the hospital. On the following day, without any previous treatment, the cornea was cauterized: slight inflammation followed. *The pupil contracted naturally*, and, in proportion as the sensibility of the eye diminished, he could distinguish objects more clearly, and at a greater distance. As this man now left the hospital contrary to the wish of the surgeons, the further progress of his case cannot be reported.

*Remarks.* This case affords an additional proof of the influence of caustic upon the iris, when applied to the cornea. It is necessary to observe, that the cornea easily recovers its transparency where it has been cauterized. In none of the above cases did there remain the slightest trace of the application of the nitrate of silver. This, indeed, is not astonishing, when we remember that this caustic will remove spots from the cornea. We lately saw a patient at Paris, whom several surgeons of the city had abandoned, the transparency of whose cornea was nearly restored by frequent cauterizations, which were directed by Professor Lallemand.

With respect to the nature of the cases we have detailed, it is evident that the third was one of hemeralopia; but the first and second must be considered as instances of amaurosis, produced by contusion of the globe of the eye.—*Revue Med.*, Août 1830.

---

### MATERIA MEDICA.

*New Mode of Preparing Medicines with Sugar.* By M. Beral. At the meeting of the Royal Academy of Medicine, on the 9th of February, 1830, M. Guibourt made a report on a new mode of preparing remedies with sugar, by M. Béral, pharmaceutist of Paris. M. Béral makes at first tinctures of the medicinal articles with alcohol and ether; these tinctures are afterwards poured upon sugar broken in small pieces; the alcohol and ether is then evaporated, and the medicinal principles which they held in solution remain with the sugar. M. B. thus obtains a medicinal sugar, which may be granulated or powdered for use.

The committee regard these preparations as very useful, and consider them as possessing the following advantages: 1st, they contain the medicinal principles isolated from the substances employed in their preparation; 2d, the medicinal principles are separated from the stimulating articles in which they were dissolved; 3d, they are soluble in water; 4th, the doses can be determined with accuracy; 5th, many of the medicinal syrups, in which the dose of the medicine is not well known, may be replaced by sugar saturated with this medicine in determined proportions; 6th, these preparations may be substituted for the oleum saccharum, used at present to give an aromatic flavor to many medicinal preparations. —*Journal Général de Médicine.*

---

MISCELLANEOUS.

St. John Long's *Liniment.* It is stated by two correspondents of the Medical Gazette, that the following liniment produces precisely the same effects as that of Mr. Long:

℞. Acid. Nitr. Muriat. ℥ij.

Ol. Terebinth. ℥i.

Ol. Camphoræ ℥v. M. fiat liniment.

Or, ℞. Acid. Nitr. Muriat. ℥ij.
Ol. Terebinth. ℥i.
Axungiæ ℥v.

Melt the lard, and then add the other ingredients, stirring the whole till quite cold.

"Either of these liniments, rubbed upon the skin with a sponge, will, in three or four minutes, cause it to be highly reddened, and the capillary vessels to be injected with blood. If the rubbing be continued, small pustules, here and there, containing a transparent limpid fluid, will make their appearance; and, if it be still persisted in, the part becomes excoriated, and an exudation of a thin clear lymph is the result. During its operation, the patients first feel warmth in the part where it is applied, then smarting, and at length actual pain."

